# Long-Term Effectiveness and Persistence of Ustekinumab in Crohn’s Disease Patients: An Observational Retrospective Study

**DOI:** 10.3390/jcm15155845

**Published:** 2026-07-27

**Authors:** Adogaye Hamid Ben Bechir, Tong Li, Ying-De Wang

**Affiliations:** Department of Gastroenterology, The First Affiliated Hospital of Dalian Medical University, Dalian 116011, China; bchirhamidben@yahoo.fr

**Keywords:** ustekinumab, Crohn’s disease, treatment outcome, inflammatory bowel disease

## Abstract

**Background/Objectives**: The incidence of Crohn’s disease (CD) is increasing, necessitating effective long-term treatments. Ustekinumab (UST) is a biologic drug that blocks interleukins (ILs) 12/23. It has been approved for moderate-to-severe CD, but real-world long-term data in Chinese populations remain limited. **Methods**: This retrospective, single-center, cohort study included patients with CD who initiated UST between June 2020 and May 2025. The primary outcomes were clinical remission at week 24 and treatment persistence through week 96. Outcomes were calculated based on the number of patients with available data at each time point. **Results**: Of 110 patients screened, 95 were included; 69.5% were bio-naïve. The clinical remission rate was 86.3% (n = 82/95) at week 24, with 80% (n = 24/30) of those patients maintaining remission at week 96. Biological remission was achieved by 51.5% (n = 17/33) at week 24, with 47.8% (n = 11/23) maintaining it through week 96. No significant difference in efficacy was observed between UST monotherapy and combination therapy with 5-ASA. Multivariate analysis revealed absence of perianal lesion and normal serum albumin were associated with clinical remission at week 24. Endoscopic remission was documented in 39.3% (n = 11/28) of reassessed patients, with a significant reduction in Simple Endoscopic Score for Crohn’s Disease on UST. Analysis of drug survival on UST revealed no statistically significant difference (*p* = 0.2044) between bio-naïve and non-naïve patients. Adverse events were reported in 9.5% of patients, with none leading to treatment discontinuation. **Conclusions**: Ustekinumab was associated with significant long-term effectiveness and a favorable safety profile, including in bio-naïve and non-bio-naïve patients.

## 1. Introduction

Crohn’s disease (CD) is a chronic inflammatory bowel disease (IBD) [1], characterized by alternating phases of clinical remission and symptomatic relapses [1,2]. In Asia, the incidence of CD has increased over time [3]. While the overall incidence in China remains lower than in Western countries, it is increasing rapidly with urbanization and socioeconomic development [4]. The current estimated incidence is 0.51–1.09 cases per 100,000 persons in China [5]. These figures are expected to increase and reach a plateau according to forecasts. Therefore, effective treatments must be available to maintain deep remission for as long as possible. Despite advancements in therapeutic strategies, the heterogeneous nature of CD marked by varying disease phenotypes, unpredictable flares, and complications necessitates lifelong management tailored to individual patient needs [6]. The advent of biologic therapies, particularly tumor necrosis factor antagonists (anti-TNF), revolutionized CD treatment by targeting specific inflammatory pathways [7]. However, primary or secondary nonresponse or adverse events lead to anti-TNF discontinuation in up to two-thirds of CD patients over time [8]. Alternative treatment approaches with different mechanisms of action, such as ustekinumab (UST), have been developed for patients who do not respond adequately to initial treatment [9].

Ustekinumab, a fully human antibody, is a biologic agent that targets the p40 subunit of interleukins 12 and 23. It has emerged as a promising therapeutic alternative by disrupting the Th1 and Th17 inflammatory pathways implicated in CD pathogenesis [10]. It was granted marketing authorization and approved for the treatment of moderate-to-severe CD in 2020 in China [11].

Several real-world studies have evaluated the effectiveness of UST in Crohn’s disease. A large systematic review and meta-analysis of 49 observational studies reported clinical remission rates of 51% at 24 weeks and 47% at 1 year [12]. Notably, real-world data suggest that the effectiveness of UST in Eastern countries may be higher than in Western countries, with clinical remission rates of 72% versus 44% at 24 weeks, respectively [12]. In China, a recent multicenter study including 102 patients reported a clinical remission rate of 87.5% at week 24 [13], while another long-term study in 348 patients reported a clinical remission rate of 70.95% at week 52 and a mean treatment survival of 172 weeks [14]. Furthermore, a meta-analysis of 63 studies demonstrated that UST had the highest 1-year persistence among advanced therapies in Crohn’s disease (77.5%), superior to TNF inhibitors [15]. However, long-term real-world data in Chinese populations remain relatively scarce [14].

These real-world findings are consistent with results from randomized controlled trials. In the UNITI program, UST was superior to placebo in inducing corticosteroid-free remission [16], with short-term efficacy of 45–55% clinical response and maintenance of remission of 53% at week 44 [17,18]. The proportion of corticosteroid-free clinical remission at week 92 was 64.4% in patients receiving UST every 8 weeks [19]. In a retrospective Japanese study, the rate of clinical remission at week 52 was 32.4% in moderate-to-severe active CD patients, with a persistence rate of 81.4% at week 104. A Dutch prospective cohort found the same figure at week 52 (35%); however, the persistence of the therapeutic effect was only 59% at week 104 [20]. Importantly, patients receiving biologic therapies in routine clinical practice may differ substantially from those enrolled in pivotal randomized controlled trials (RCTs). Indeed, only a minority of real-world patients with inflammatory bowel disease would qualify for corresponding RCTs, raising questions about the external validity of trial results [21]. Real-world studies have shown that biologics demonstrate similar effectiveness and safety in eligible and non-eligible patients, underscoring the importance of real-world evidence to complement RCT findings. More studies are needed to clarify the situation of the persistence of the long-term therapeutic effect of UST.

This retrospective observational study investigates the short-term effectiveness and long-term persistence of UST in a real-world cohort of patients with CD. Our findings aim to help clinical decision-making for a complex, chronic condition demanding personalized care [22].

## 2. Materials and Methods

### 2.1. Study Design

This was a retrospective observational and analytical single-center cohort study conducted at the First Affiliated Hospital of Dalian Medical University, China.

### 2.2. Population

**Figure 1:**
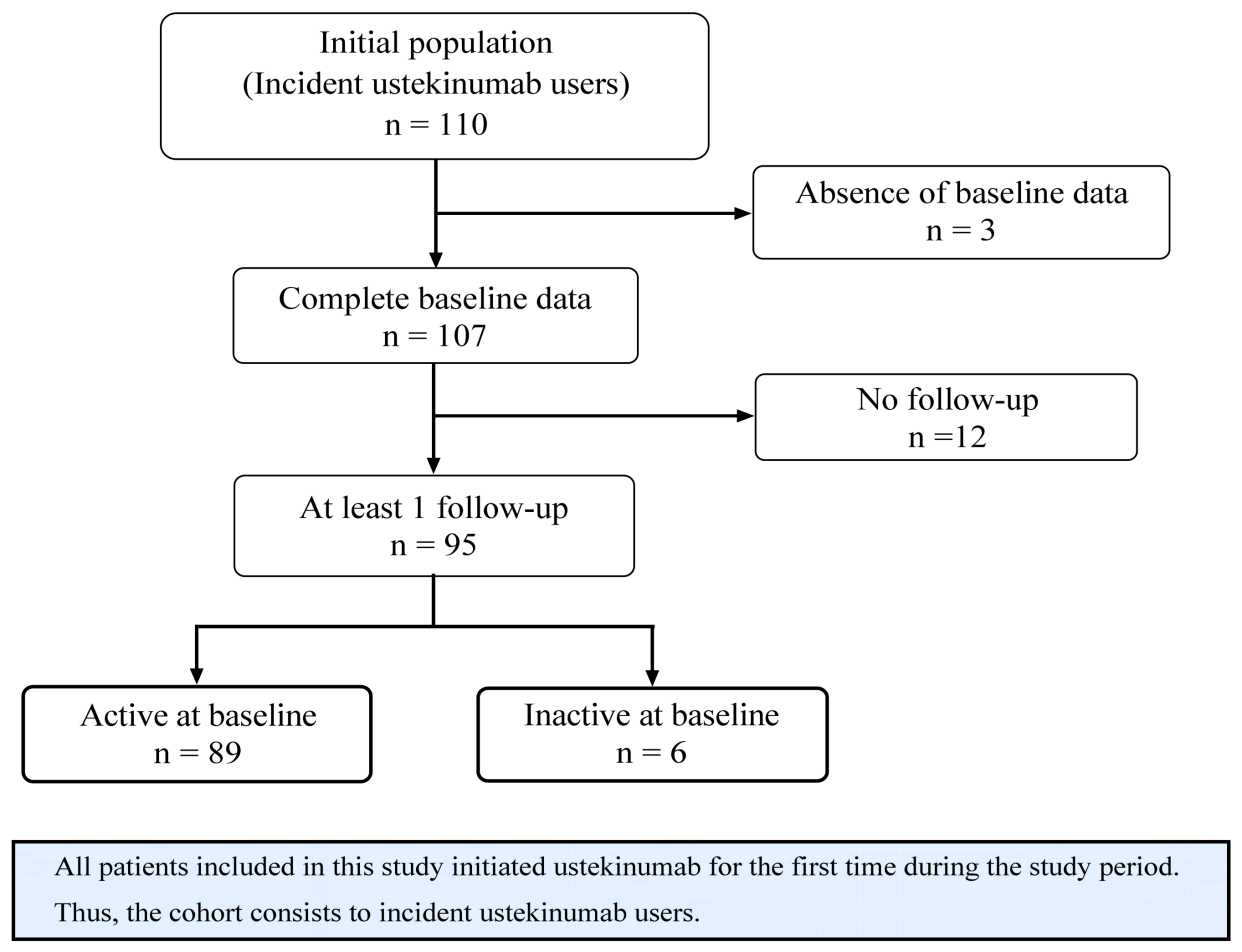
Flowchart of patient selection.

A list of all patients with a diagnosis of CD who received UST was obtained. All those who received UST regardless of previous treatment and had at least one follow-up were included. We have excluded patients with insufficient or absent baseline data. All included patients were incident users of UST, defined as having received their first-ever dose of UST during the study period. The initial intravenous dose of UST was administered according to weight as per the label (<55 kg: 260 mg; 55–85 kg: 390 mg; >85 kg: 520 mg). The following doses were administered at 8- or 12-week intervals, based on the discretion of the treating physician. Patients who had an initial response or remission at 24 weeks were continued on the treatment for maintenance. Figure 1 shows the flowchart of patient selection.

### 2.3. Data Collection

All data were extracted retrospectively from the electronic database. We collected at baseline: sociodemographic data; history of disease; clinical data (age at the diagnosis, behavior and localization according to the Montreal classification, Harvey–Bradshaw Index (HBI), extra-intestinal manifestations (EIM)); biological variables (fecal calprotectin (fCal), C-reactive protein (CRP), hemoglobin (Hb), albuminemia (Alb), erythrocyte sedimentation rate (ESR), platelets (PLT), white blood cells (WBC)); Simple Endoscopic Score for Crohn’s Disease (SES-CD). Data were collected every 8 weeks after the initiation of UST until 1 year, then every 6 months.

### 2.4. Outcomes

The primary outcome was the proportion of patients with clinical remission at week 24 and persistence at week 96. The secondary outcomes included: clinical response, biological remission, endoscopic remission, safety, predictive factors of remission at 24 weeks and persistence of remission at week 96. Follow-up duration was defined as the period from the first dose of UST until the end of follow-up or discontinuation of treatment. Patients who required dose optimization, re-induction, or interval shortening were considered to have experienced treatment failure and classified as non-responders. This definition was chosen because these interventions represent a clinical indicator of suboptimal therapeutic response, reflecting the need for treatment intensification in routine practice. This approach is consistent with previous real-world studies evaluating biologic persistence in IBD [23,24]. Patients who remained on UST but required dose optimization, re-induction, or interval shortening were censored at the time of respective intervention for persistence analysis. The proportions were calculated depending on the number of data available at each time point.

### 2.5. Definitions of Terms

Montreal classification was used to classify the age at diagnosis (A), the location of the disease (L), and its behavior (B). Clinical response was defined as a reduction in HBI score of ≥3 points compared to baseline. It only concerns patients with clinically active disease at baseline (HBI ≥ 5 points). Clinical remission was defined as HBI ≤ 4 [2,25]. Biological remission was defined as a CRP serum concentration < 5 mg/L [2]. Endoscopic remission was assessed using SES-CD and was classified: <3 inactive, 3–6 Mild activity, 7–15 moderate activity, >15 Severe activity [25]. Treatment persistence was defined as the time from the first UST dose to treatment discontinuation. Discontinuation was defined as physician-directed cessation (including loss of response or adverse events) or switching to another biologic. Patients were censored at their last known UST administration date if they remained on treatment or were lost to follow-up.

### 2.6. Statistical Analysis

Quantitative and qualitative data were summarized as medians with interquartile ranges (IQR) and counts (percentages). Group comparisons used Fisher’s exact or chi-squared tests for categorical variables. For continuous variables, comparisons between multiple groups were conducted using a one-way analysis of variance (ANOVA). The assumption of homogeneity of variances was systematically assessed for each ANOVA using Bartlett’s test. When this assumption was violated (*p* < 0.05), the results of the standard ANOVA were interpreted with caution, and the more robust Brown–Forsythe test was reported alongside it. In such cases, post-hoc comparisons were performed using the Games-Howell test, which does not assume equal variances, to identify specific group differences. For comparisons involving only two groups, Student’s *t*-test or the Mann–Whitney U test was applied as appropriate. Predictive variables were assessed by univariate and multivariate analyses using logistic regression. Variables with a *p*-value of less than 0.1 in the univariate analysis were included in a multivariate model using backward stepwise logistic regression. The Kaplan–Meier method assessed the probability of clinical remission failure and the probability of UST persistence over 96 weeks. Statistical significance for all analyses was defined as a two-sided *p*-value of less than 0.05. All statistical analyses were performed using SPSS software version 29 and GraphPad Prism version 9.5.1. This study is reported in accordance with the STROBE Statement for observational studies (Supplementary Table S1).

### 2.7. Ethical Considerations

The study was conducted in accordance with the Declaration of Helsinki and approved by the Ethics Committee of the First Affiliated Hospital of Dalian Medical University (Approval number: PJ-KS-KY-2025-1040; Date: 7 November 2025).

## 3. Results

### 3.1. Baseline Characteristics

A total of 110 records of patients with CD who received UST were analyzed. Fifteen records were excluded: three for lack of baseline data and twelve for lack of at least one follow-up data point after induction. Among the remaining 95 patients, 89 had active disease at the time of induction. Clinical remission was determined in both the total and active populations, while clinical response was only determined in the active population. A total of 89 (93.7%) had active disease at baseline, with 67 (75.3%) moderate-to-severe. The minimum follow-up period was three weeks. The available data were sufficient for inclusion in the effectiveness analysis. The median follow-up duration was 75.6 weeks (95% CI 41–132.1). Because patient assessments were performed at the attending physician’s discretion, not all patients had complete data at every time point. Therefore, we based our outcome assessments on the number of available data at each endpoint. Baseline characteristics are shown in Table 1. Additional baseline characteristics stratified by prior biologic exposure are presented in Supplementary Table S2.

### 3.2. Clinical Response

**Figure 2:**
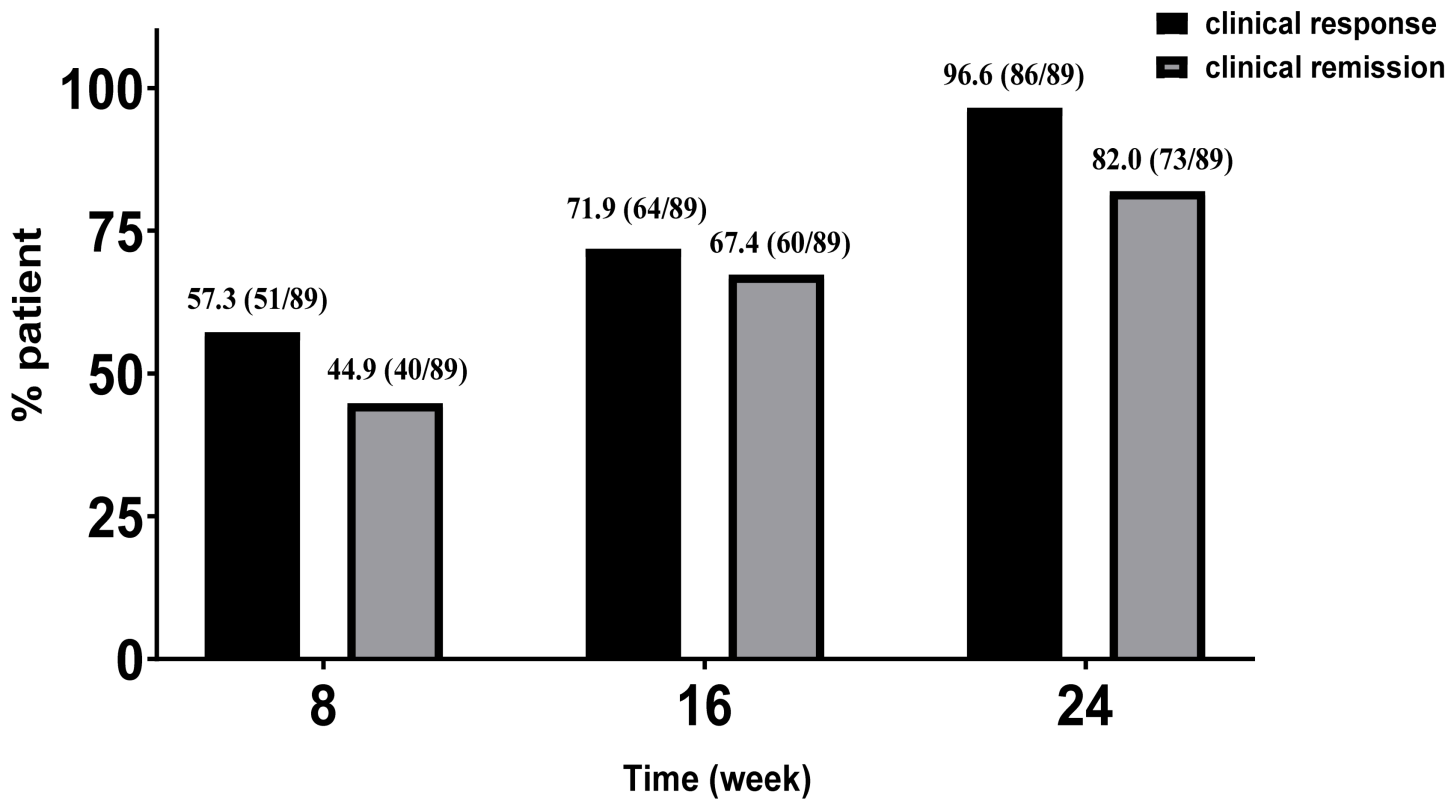
Rates of cumulative proportion of clinical response and remission over time.

At week 24, a cumulative proportion of 96.6% (n = 86/89) of active patients achieved clinical response over a median duration of 8.3 (7.7–14) weeks. The cumulative proportion of patients achieving clinical response at 8 and 16 weeks was 57.3% (n = 51/89) and 71.9% (n = 64/89) (Figure 2).

### 3.3. Clinical Remission

**Figure 3:**
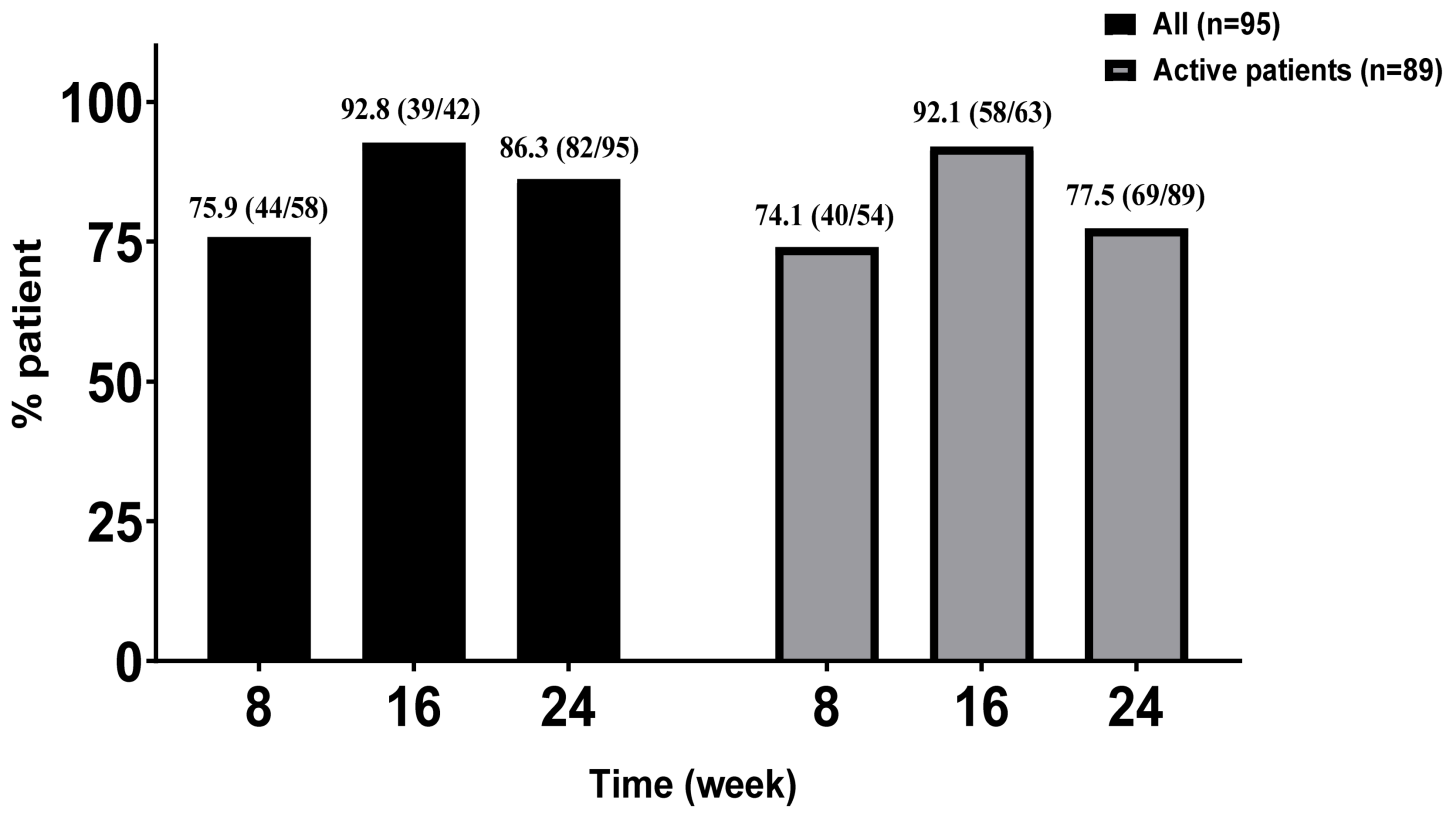
Proportion of clinical remission over time in all active patients.

The proportion of patients achieving remission was 75.9% (n = 44/58) at week 8, rising to 92.8% (n = 39/42) at week 16, and was 86.3% (n = 82/95) by week 24. Among patients who had active disease at baseline, clinical remission rates followed a similar trend, reaching 74.1% (n = 40/54) at week 8, 92.1% (n = 58/63), and 77.5% (n = 69/89) at weeks 16 and 24, respectively (Figure 3).

**Figure 4:**
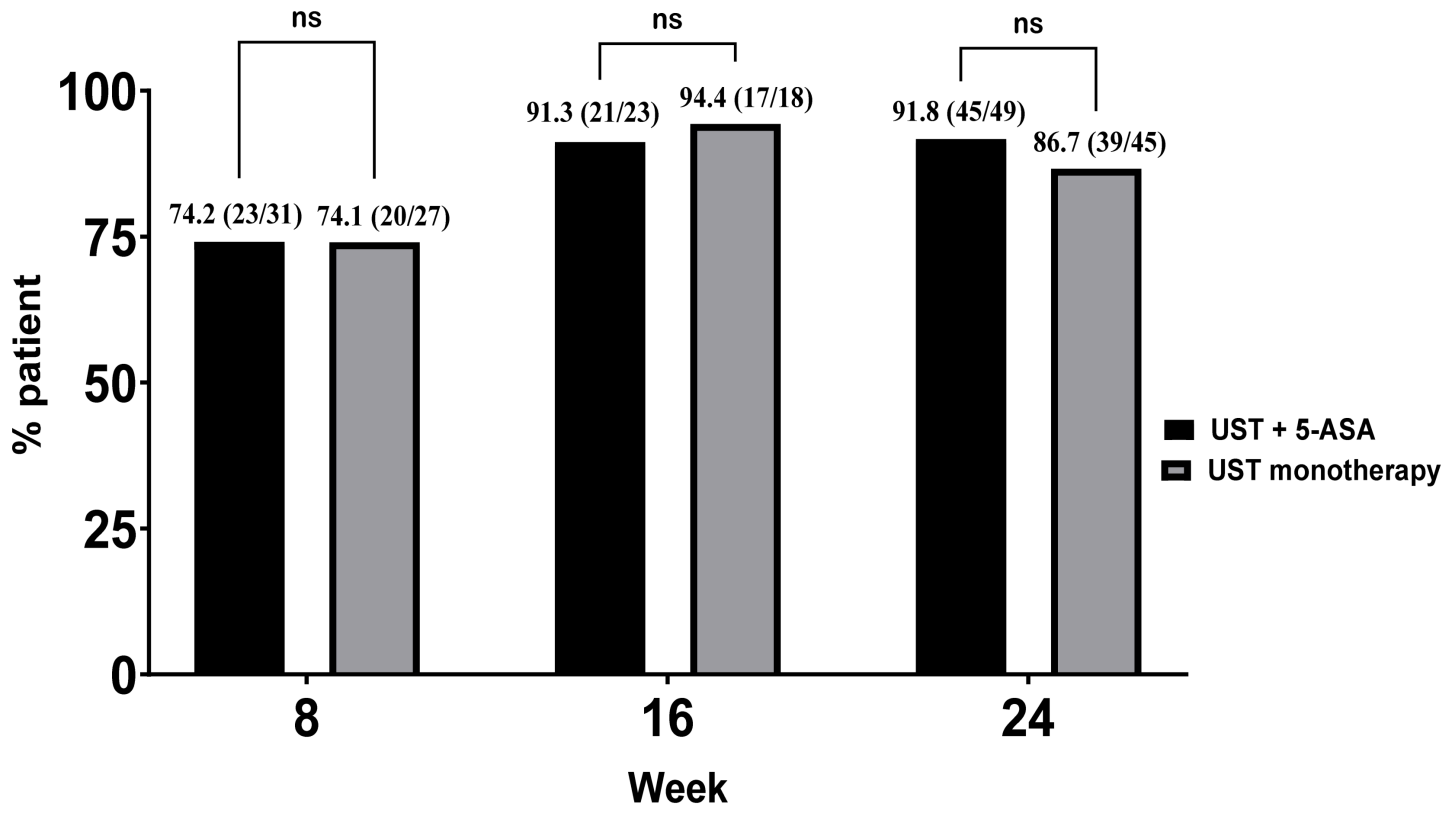
Proportion of clinical remission in patients receiving UST in combination with 5-ASA compared to UST monotherapy. ns, non-significant.

In patients receiving UST in combination with 5-ASA, clinical remission was 74.2% (n = 23/31), 91.3% (n = 21/23), and 91.8% (n = 45/49) at 8, 16, and 24 weeks, respectively. It was similar at the same time points in patients who did not receive 5-ASA (Figure 4).

### 3.4. Treatment Persistence

**Figure 5:**
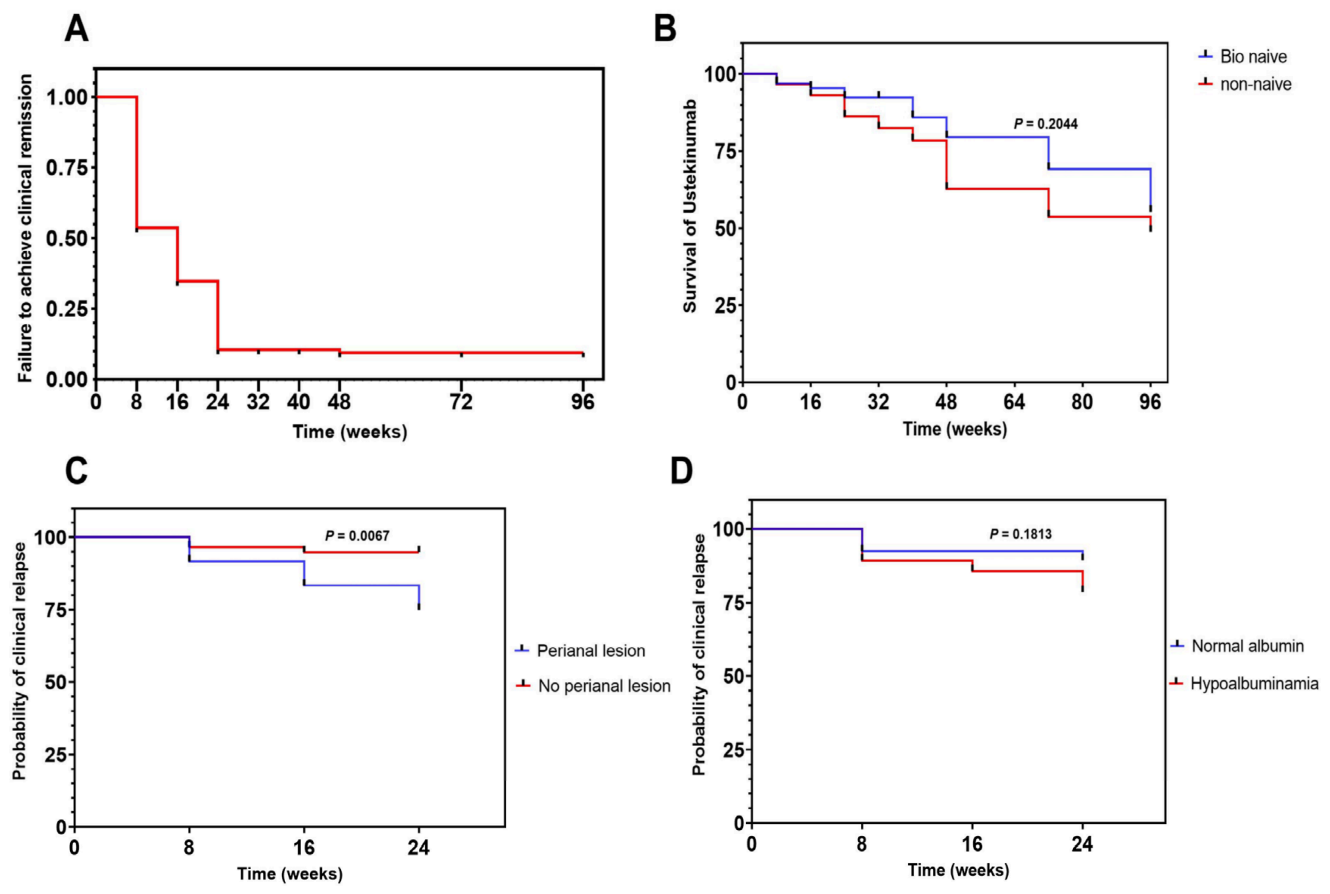
The Kaplan–Meier curves show the probability of (**A**) failure to achieve clinical remission, (**B**) survival of UST among bio-naïve and non-bio naïve, (**C**) clinical relapse in patients with vs. without perianal lesion, (**D**) clinical relapse in patients with normal serum albumin vs. hypoalbuminemia.

Of the 82 patients in clinical remission at week 24, 80% (n = 24/30) maintained it at 96 weeks. The proportions of patients in clinical remission at weeks 32, 40, 48, and 72 were 94.6% (n = 35), 81.5% (n = 22), 88.6% (n = 39), and 86.1% (n = 31), respectively. Among the baseline active patients (n = 89) who achieved clinical remission at 24 weeks, 69.4% remained on UST at week 96. The Kaplan–Meier curve (Figure 5A) shows that some patients achieved clinical remission even after many months of therapy, supporting the use of UST for long-term maintenance.

Analysis of the probability of remaining on UST therapy revealed no statistically significant difference (*p* = 0.2044) between bio-naïve and non-naïve patients, despite visible separation in the Kaplan–Meier curves (Figure 5B). While bio-naïve patients demonstrated a consistent trend toward superior long-term treatment maintenance, this observed difference did not reach statistical significance within the study parameters.

Analysis of the probability of clinical relapse over 24 weeks indicates that patients presenting with perianal lesions are at a significantly higher risk of relapse compared to those without such lesions (*p* = 0.0067) (Figure 5C). In contrast, there is no statistically significant difference (*p* = 0.1813) between patients with normal serum albumin levels and those with hypoalbuminemia (Figure 5D).

Stratified analyses of treatment persistence by disease behavior are provided in Supplementary Figure S1.

### 3.5. Biological Remission

Biological remission was assessed using CRP. Among patients with CRP at baseline (n = 104), only 79 had active CRP (≥5 mg/L). The proportion of patients in biological remission was 52.2% (n = 24/46), 48.5% (n = 16/33), 51.5% (n = 17/33), and 47.8% (n = 11/23) at 8, 16, 24, and 96 weeks, respectively.

In the subgroup of 69 patients with clinical baseline activity (HBI ≥ 5) and active CRP (≥5 mg/L), biological remission rates were 53.5% (n = 23/43), 43.7% (n = 14/32), 48.4% (n = 15/31), and 50% (n = 11/22), at 8, 16, 24, and 96 weeks, respectively.

**Figure 6:**
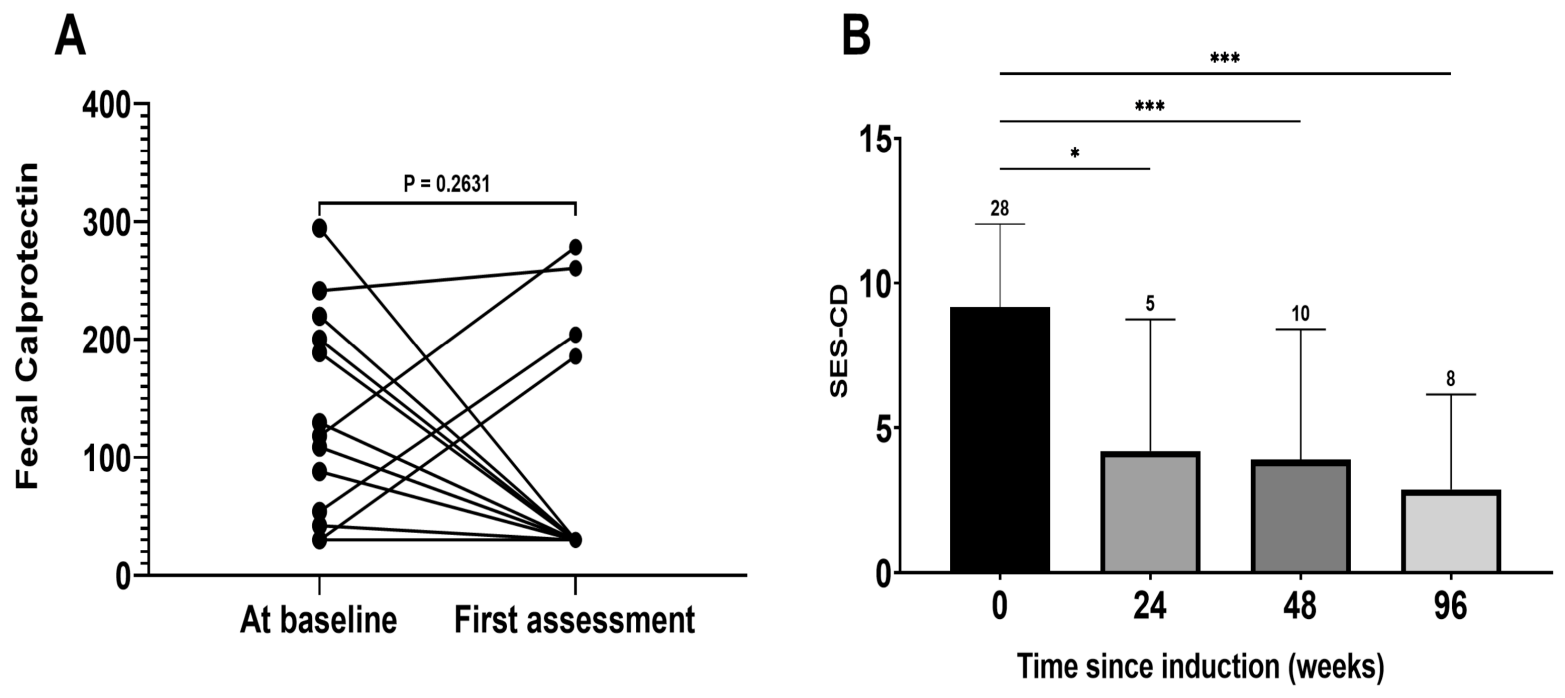
This figure shows (**A**) the fecal calprotectin at baseline and at the first evaluation after initiation of UST in 13 patients, (**B**) evolution of SES-CD over a period of 96 weeks. * *p* < 0.05 (compared with baseline). *** *p* < 0.01 (compared with baseline). SES-CD, Simple Endoscopic Score for Crohn’s Disease.

Thirty-eight (38) patients had a fecal calprotectin measurement at baseline. Of these, thirty-three had a value above the normal range (≥30 µg/g), and only four had a value ≥ 250 µg/g. Only 13 patients had at least one assessment after the induction of UST. The comparison of fecal calprotectin levels between baseline and the first assessment shows no statistically significant difference (*p* = 0.2631) (Figure 6A).

As shown in Table 2, CRP decreased significantly at week 8 in both the general and active population, then increased slightly while remaining within the normal range. Improvement in biological parameters was also observed throughout the follow-up with regard to Alb and Hb values.

### 3.6. Endoscopic Remission

At treatment initiation, 65.3% (62/95) patients had endoscopic data, of which 64.2% (61/95) were active (SES-CD ≥ 3). Twenty-eight (45.9%) patients among those with endoscopic activity underwent endoscopic reassessment. Endoscopic remission was achieved after a median duration of 25.6 weeks by 39.3% (11/28) of patients. The SES-CD score showed a marked decrease following treatment initiation, with comparisons to baseline revealing statistically significant reductions (*p* < 0.05) that were maintained throughout the long-term follow-up. Figure 6B shows the evolution of SES-CD over a period of 96 weeks.

### 3.7. Optimization, Re-Induction and Adverse Events

Nine (9) cases of adverse events (AEs) were recorded. Among these, five cases of infection were recorded, three of which required hospitalization for intravenous antibiotic therapy. One patient developed an intestinal perforation at week 27 and was treated surgically. Fifteen participants underwent reinduction of intravenous UST with the same initial dose, seven of whom had previously had their UST maintenance dose optimized.

### 3.8. Predictors of Clinical Remission

The factors associated with clinical remission at 24 and 96 weeks are presented in Table 3. In univariate analysis, disease duration ≥ 3 years (OR: 0.4 [95% CI: 0.1–1]) and absence of perianal lesion (OR: 0.1 [95% CI: 0.03–0.5]) were associated with a higher likelihood of achieving clinical remission at week 24. A normal albumin level (OR: 0.5 [95% CI: 0.2–1]) showed a borderline association. In multivariate analysis, absence of perianal lesion and normal serum albumin (≥35 g/L) remained independent predictors of clinical remission at 24 weeks of treatment.

In the analysis of the active patient population, no baseline factor demonstrated a significant association with clinical remission at weeks 24 and 96.

In univariate analysis, no factor was found to be associated with biological remission at weeks 24 and 96.

## 4. Discussion

To our knowledge, this is among the first real-world studies to evaluate the long-term (96-week) effectiveness and persistence of ustekinumab in a Chinese cohort of patients with Crohn’s disease. Beyond its geographic setting, our study contributes specific evidence on treatment durability, identifies clinically relevant predictors of remission, and examines the utility of concomitant 5-ASA, all of which address knowledge gaps not yet covered by existing short-term or Western-dominant real-world data. These findings complement existing real-world data from Western and Japanese cohorts.

The demographic profile of our cohort, comprising a high proportion of bio-naïve patients, offers a valuable perspective complementary to the existing literature, which often focuses on anti-TNFα-experienced populations. The cumulative clinical response rates were 57.3% at week 8, 71.9% at week 16, and 96.6% at week 24. These findings highlight the delayed response phenomenon associated with UST. This gradual improvement aligns with findings from other real-world studies and can be attributed to factors such as younger age, absence of corticosteroids at induction, a history of EIMs, and isolated ileal disease [13,26]. The slow onset of action may be further explained by the pharmacokinetic profile of UST, which requires time to reach steady-state concentrations [27]. This pattern underscores the importance of allowing an adequate trial period before deeming UST ineffective.

Our week 8 clinical response rate (57.3%) appears higher than the 33.5% and 37.8% reported in the UNITI-1 trial in patients receiving intravenous UST at a dose of 130 mg or approximately 6 mg/kg, respectively [17]. This difference is likely not due to superior efficacy but rather to fundamental differences in study populations. The UNITI trials exclusively enrolled patients with moderate-to-severe disease who had failed or were intolerant to anti-TNFα therapy, a group typically more refractory to subsequent biological interventions. Our cohort’s higher bio-naïve prevalence may explain the more robust initial response, a finding supported by other real-world cohorts [28]. However, our long-term remission rate at week 96 (80%) is highly consistent with the 92-week results from the IM-UNITI trial (70.4–76.4%), which consisted of participants who had failed or were intolerant to steroids and/or immunosuppressants, suggesting that while initial response may vary by population, long-term remission rates converge favorably [29]. This reinforces the concept that UST is an effective long-term therapeutic strategy across diverse patient phenotypes.

A key finding of our study is the lack of significant difference in clinical remission rates between patients on UST monotherapy and those on combination therapy with 5-ASA. At all assessed time points, remission rates were similar. This observation is supported by a growing body of evidence which indicates that 5-ASA provides no synergistic benefit when combined with advanced biologics such as UST [30,31]. This practice has been increasingly challenged, as position papers from major international societies state there is no evidence to support the use of 5-ASAs for the treatment of CD itself [32]. The practice of continuing 5-ASA alongside biologics may be rooted in historical convention or a desire for a multi-mechanistic approach, particularly in bio-naïve patients [30,33]. However, our data, in concert with others, question the utility of this strategy, potentially supporting de-escalation to monotherapy to reduce pill burden and cost without compromising efficacy.

Biomarker analysis revealed sustained biological remission, with 52.2% of patients achieving normalized CRP at week 8, and nearly half maintaining this response through week 96. This was accompanied by significant improvements in nutritional parameters such as albumin and hemoglobin, indicating a reduction in systemic inflammation and associated malnutrition. By effectively inhibiting the IL-12/23 pathway, UST mitigates gut inflammation, thereby restoring intestinal integrity and improving nutrient absorption [34,35]. While our CRP-based remission rates are numerically higher than the biochemical remission (defined by both CRP ≤ 10 mg/L and fcal ≤ 200 µg/g) reported by Thomas et al. [20], the differing definitions and timepoints preclude a direct comparison. CRP, while widely used and correlated with disease activity, is a systemic acute-phase reactant that may not fully reflect intestinal mucosal inflammation, particularly in patients with isolated ileal disease or those with normal baseline CRP [14,36]. fCal is a useful and more specific biomarker of intestinal inflammation that not only predicts clinical and endoscopic remission but also identifies subjects at risk of relapse [37,38]. Although its cut-off is subject to debate, it remains a reliable non-invasive tool. Its limited use in our study is an acknowledged constraint. Furthermore, real-world studies have confirmed that higher UST trough concentrations are associated with improved biomarker and endoscopic outcomes [39], a potential area for future research in our population.

Our results, illustrated by the Kaplan–Meier curve, indicate that UST is an effective and durable treatment for inducing and maintaining clinical remission in patients with CD. The response kinetics in our real-world cohort align remarkably with phase 3 trial data. The IM-UNITI study reported that approximately 55% of patients receiving UST every 8 weeks were in clinical remission at week 44, a rate sustained through week 96 [17]. Our data, showing a cumulative probability of approximately 75% for achieving remission at two years, are consistent with this yet suggest potentially higher efficacy in clinical practice. This difference may be attributed to the inclusion of more refractory populations in clinical trials and is corroborated by large observational studies, such as the Dutch ICC registry, which reported a treatment persistence rate exceeding 80% at one year [23].

Notably, this durable efficacy supports the use of UST as a robust long-term maintenance therapy. The sustained response can be largely attributed to its low immunogenicity. Unlike anti-TNF agents, where the formation of anti-drug antibodies is a common cause of secondary loss of response, UST’s favorable immunogenic profile [39] facilitates prolonged drug persistence. This pharmacological advantage is further evidenced by clinical data from Ma et al. (2017), where a majority of patients maintained their response over several years [29].

Despite this overall high effectiveness, our study supports that a subset of patients who initially achieve remission experience a loss of response by year two. Therefore, identifying predictors of non-response is a critical clinical objective. The literature indicates that factors such as a history of failure to multiple biologic classes, active penetrating disease, or severe ileal involvement are associated with poorer outcomes [40]. These observations underscore the importance of proactive therapeutic drug monitoring, including the assessment of serum UST levels, to promptly identify patients who may benefit from dose optimization or a switch to an alternative therapeutic class.

When we compare the survival probability of UST in bio-naïve and non-naïve patients, no significant difference emerges. Although the literature suggests superior efficacy of UST in patients who have not received any biologic therapy, our results, along with some studies, could reflect the robust efficacy of UST even in patients who have already experienced biologics. Lee et al., in a Korean cohort, did not identify anti-TNF status as an independent predictor of UST efficacy [41]. Similarly, long-term analyses in CD have reported that the benefits of maintenance therapy can be durable and comparable across subgroups, with differences that are not always statistically significant for all endpoints [42,43].

The safety profile of UST in this Chinese cohort was highly favorable, with a low incidence of AEs and hospitalization, and no events leading to treatment discontinuation. This aligns with the established safety data from global clinical trials and other real-world studies in Asian populations [13], and is further supported by large pooled safety analyses [18], supporting that UST is a well-tolerated long-term treatment option.

Our multivariate analysis identified two major independent predictors of clinical remission at week 24. The perianal lesions, a feature typically associated with more severe disease and a diminished response to biological therapies, including anti-IL-12/23 agents, was a strong negative predictor [44]. Concurrently, a normal serum albumin level was significantly associated with an increased probability of remission, reinforcing the role of albumin as a robust marker of systemic inflammation and nutritional status. Hypoalbuminemia has been identified as a predictor of poor response, requiring close monitoring of clinical activity and potentially optimization of treatment doses [45]. Furthermore, a non-significant trend toward higher remission rates was observed when UST was used as a first-line therapy, aligning with the recognized benefit of early intervention. Collectively, these factors reflecting disease phenotype, inflammatory burden, and nutritional status could form the basis for a predictive score to identify patients who may benefit from treatment intensification or closer monitoring.

Several limitations inherent to our retrospective, single-center design must be acknowledged. The variability in follow-up intervals, influenced by economic factors, combined with the inclusion of patients who had not yet completed the full follow-up period, led to missing data at certain time points and reduced the sample size for the long-term analyses. We utilized an as-observed analysis, which may inflate efficacy estimates compared to strict intention-to-treat approaches. Furthermore, objective markers of mucosal healing were incomplete: endoscopic data, the gold standard for mucosal healing, were not routinely available for all patients, and fecal calprotectin, a more sensitive and specific marker of intestinal inflammation than CRP, was measured in only a minority. While CRP normalization was observed in approximately half of the patients, this may underestimate the true rate of mucosal healing, as CRP can remain normal in patients with ongoing intestinal inflammation [46]. This limitation is particularly relevant in our cohort, where the observed CRP-based biological remission rate (51.5%) stands in contrast to the limited fCal data available. This discrepancy highlights the potential for CRP to underestimate the true burden of mucosal inflammation, and underscores the need for future studies to incorporate fCal as a standard monitoring tool. Finally, multivariate findings should be interpreted with caution and considered exploratory and hypothesis-generating rather than confirmatory.

Despite these limitations, our study significantly strengthens the real-world evidence for UST’s long-term efficacy and safety in managing CD, particularly within a Chinese population with a high proportion of bio-naïve patients. We add to the growing body of evidence that the absence of a clinical benefit for combining 5-ASA with UST, a practice that should be re-evaluated in light of current evidence [32]. Future prospective, multi-center studies with standardized follow-up, systematic endoscopic evaluation, and therapeutic drug monitoring are warranted to validate these findings and further explore predictors of long-term response [39].

## 5. Conclusions

Overall, UST was associated with significant long-term effectiveness and a favorable safety profile in this real-world cohort, with most patients maintaining clinical remission through 96 weeks. Our findings support the use of UST as a durable treatment option for CD, including in bio-naïve and bio-experienced patients.

## Figures and Tables

**Table 1:** Baseline characteristics.

Variables	Total Study Population (N = 95)	Patients with Clinical Disease Activity at Baseline (N = 89)
Age (years)	32 (23–48)	33 (23–48)
Sex, male	60 (63.2)	57 (64)
Disease duration (years)	2 (0.65–4)	2 (0.6–4)
Age at diagnosis †		
A1	7 (7.4)	7 (7.9)
A2	61 (64.2)	56 (62.9)
A3	27 (28.4)	26 (29.2)
Location †		
L1 ileal	11 (11.6)	9 (10.1)
L2 colonic	24 (25.3)	22 (24.7)
L3 ileocolonic	60 (63.2)	58 (65.2)
Upper GI tract	5 (5.3)	5 (5.3)
Behaviour †		
B1	45 (47.4)	43 (48.3)
B2 stricturing	42 (44.2)	38 (42.7)
B3 penetrating	8 (8.4)	8 (9)
Perianal disease	37 (38.9)	34 (38.2)
Prior surgery		
Perianal	19 (20)	19 (21.4)
Intestinal	15 (15.8)	12 (13.5)
Extra-intestinal manifestations	10 (10.5)	8 (9)
Previous steroid use	8 (8.4)	8 (9)
Bio-naïve	66 (69.5)	64 (71.9)
Prior anti-TNF		
≥1	21 (22.1)	18 (20.2)
≥2	2 (2.1)	2 (2.2)
Prior Vedolizumab	4 (4.2)	3 (3.4)
Concomitant medication with UST		
Mesalazine	51 (53.7)	49 (55.1)
Azathioprine	1 (1.1)	1 (1.1)
Disease activity		
HBI	8 (7–11)	9 (8–11)
CRP (mg/L)	16.64 (5.3–43.2)	20.6 (7.7–43.3)
SES-CD	9.7 (7–12)	9 (7–12)
Other tests		
WBC	6.9 (5.2–8.8)	6.9 (5.2–8.9)
Hb	11.7 (9.9–13.1)	11.5 (9.9–12.9)
PLT	302 (221.5–358.5)	312 (225–359)
Alb	37 (32.1–40.9)	38.1 (32.1–41)
ESR	28.5 (15–49)	32 (16–51)

† Phenotypes were categorized using the Montreal classification system. The values are described as number (%) or median (IQR). Alb, Albumin; CRP, C-reactive protein; ESR, Erythrocyte Sedimentation Rate; Hb, Hemoglobin; HBI, Harvey–Bradshaw Index; PLT, Platelets; SES-CD, Simple Endoscopic Score for Crohn’s Disease; WBC, White Blood Cells.

**Table 2:** Biological outcomes over time.

	Week 0	Week 8	Week 24	Week 96
CRP ^1^, median (IQR), mg/L	16.3 (5.3–43.2)	3.2 (2.8–9.2) *	3.9 (1.5–9.2) **	4.5 (0.5–20.8)
CRP ^2^, median (IQR), mg/L	20.6 (7.7–43.3)	3.3 (2.5–9.2) **	4.9 (1.5–47.6) **	4.5 (0.5–19.5) **
Hb, median (IQR), g/dL	11.7 (9.9–13.1)	12.7 (10.9–13.9)	13.6 (12.1–14.4) **	13.6 (11.9–14.7) **
Alb, median (IQR), g/L	37 (32.1–40.9)	42.2 (39.8–44.7)	43 (41.5–46.2) **	42 (38.8–46.2) **
ESR, median (IQR), mm/h	28.5 (15–49)	16.5 (10–39)	14.5 (5–34)	29.5 (16–62)

^1^ CRP at baseline. ^2^ CRP of active patients. * *p* < 0.05 (compared with baseline). ** *p* < 0.01 (compared with baseline). Alb, Albumin; CRP, C-reactive protein; ESR, erythrocyte sedimentation rate; Hb, Hemoglobin; IQR, interquartile range.

**Table 3:** Predictors of clinical remission by univariate and multivariate analysis.

Factors	Univariate		Multivariate	
	*p*	OR (95% CI)	*p*	OR (95% CI)
**Week 24**				
Gender, male	0.6	0.7 (0.2–2.5)		
First line UST (Bio-naïve)	0.1	2.5 (0.6–9.5)		
Disease duration ≥ 3 years	0.04	0.4 (0.1–1)	0.32	2.4 (0.4–13.7)
Age at diagnosis				
A1 ≤ 16 years		Reference		
A2 17–40 years	0.8	1.2 (0.1–12.3)		
A3 > 40 years	0.7	0.7 (0–7.5)		
Disease location				
L1 ileal		Reference		
L2 colonic	0.8	0.8 (0.1–5.2)		
L3 Ileocolonic	0.4	2 (0.3–11.4)		
Disease behavior				
B1 Inflammatory disease		Reference		
B2 stricturing	0.2	0.4 (0.1–1.8)		
B3 penetrating	0.2	0.2 (0–1.9)		
Absence of perianal lesion	<0.001	0.1 (0.03–0.5)	0.002	17.6 (2.85–109.0)
Combination therapy with 5-ASA	0.6	0.7 (0.2–2.5)		
Albuminemia ≥ 35 g/L	0.05	0.5 (0.2–1)	0.04	2.6 (1.05–6.53)
ESR < 20 mm/H	0.08	0.5 (0.2–1.1)		
WBC 4–10 × 10^9^/L	0.4	1.7 (0.4–6.3)		
Hemoglobin 10–12.99 g/dL	0.1	0.5 (0.2–1.1)		
Platelet 150–450 × 10^9^/L	0.5	0.6 (0.1–2.8)		
**Week 96**				
First-line UST (Bio-naïve)	0.8	1.2 (0.1–8.2)		
Disease duration ≥ 3 years	0.5	0.6 (0.1–3.6)		
Absence of perianal lesion	0.4	0.5 (0–3)		
Albuminemia ≥ 35 g/L	0.8	1.1 (0.2–4.5)		
ESR < 20 mm/H	0.1	0.4 (0.1–1.3)		
Hemoglobin 10–12.99 g/dL	0.8	1 (0.3–3.8)		

ESR, erythrocyte sedimentation rate; WBC, white blood cells.

## Data Availability

Datasets generated and/or analyzed during the current study are available from the corresponding author on reasonable request.

## Supplementary Materials

The following supporting information can be downloaded at: https://www.mdpi.com/article/10.3390/jcm15155845/s1, Figure S1: Kaplan–Meier curves for treatment persistence stratified by disease behavior; Table S1: STROBE Statement—Checklist of items that should be included in reports of cohort studies, Table S2: Baseline characteristics stratified by bio-naïve versus bio-experienced status.

## Abbreviations

The following abbreviations are used in this manuscript:

Alb	Albumin
AZA	Azathioprine
CD	Crohn’s disease
CRP	C-reactive protein
ESR	Erythrocyte Sedimentation Rate
EIM	Extra-Intestinal Manifestation
fCal	Fecal calprotectin
Hb	Hemoglobin
HBI	Harvey–Bradshaw Index
IBD	Inflammatory bowel disease
5-ASA	Mesalazine
PLT	Platelets
SES-CD	Simple Endoscopic Score for Crohn’s Disease
Anti-TNF	Tumor Necrosis Factor antagonists
UST	Ustekinumab
WBC	White Blood Cells
