# Supplementary material for: Long-Term Effectiveness and Persistence of Ustekinumab in Crohn’s Disease Patients: An Observational Retrospective Study
Source: J Clin Med. 2026 Jul 27;15(15):5845. doi: 10.3390/jcm15155845 (PMC13466482; doi:10.3390/jcm15155845)
Supplement: Supplementary file 1 [file jcm-15-05845-s001.zip › jcm-4347097-supplementary.pdf]

Supplementary Table S1. STROBE Statement - Checklist of items that should be included in reports of ***cohort studies***

	Item No	Recommendation	Page No
**Title and abstract**	1	(*a*) Indicate the study's design with a commonly used term in the title or the abstract	Page 1
		(*b*) Provide in the abstract an informative and balanced summary of what was done and what was found	Page 1
**Introduction**			
Background/rationale	2	Explain the scientific background and rationale for the investigation being reported	Page 1-2
Objectives	3	State specific objectives, including any prespecified hypotheses	Page 2
**Methods**			
Study design	4	Present key elements of study design early in the paper	Page 3
Setting	5	Describe the setting, locations, and relevant dates, including periods of recruitment, exposure, follow-up, and data collection	Page 3
Participants	6	(*a*) Give the eligibility criteria, and the sources and methods of selection of participants. Describe methods of follow-up	Page 3 & page 4
		(*b*) For matched studies, give matching criteria and number of exposed and unexposed	N.A.
Variables	7	Clearly define all outcomes, exposures, predictors, potential confounders, and effect modifiers. Give diagnostic criteria, if applicable	Page 4
Data sources/ measurement	8*	For each variable of interest, give sources of data and details of methods of assessment (measurement). Describe comparability of assessment methods if there is more than one group	Page 3-4
Bias	9	Describe any efforts to address potential sources of bias	Page 4 & page 13
Study size	10	Explain how the study size was arrived at	Page 3
Quantitative variables	11	Explain how quantitative variables were handled in the analyses. If applicable, describe which groupings were chosen and why	Page 4
Statistical methods	12	(*a*) Describe all statistical methods, including those used to control for confounding	Page 4
		(*b*) Describe any methods used to examine subgroups and interactions	Page 5
		(*c*) Explain how missing data were addressed	Page 4
		(*d*) If applicable, explain how loss to follow-up was addressed	Page 4
		(*e*) Describe any sensitivity analyses	N.A.
**Results**			
Participants	13*	(a) Report numbers of individuals at each stage of study—eg numbers potentially eligible, examined for eligibility, confirmed eligible, included in the study, completing follow-up, and analysed	Page 6
		(b) Give reasons for non-participation at each stage	Page 6
		(c) Consider use of a flow diagram	Page 4

Descriptive data	14*	(a) Give characteristics of study participants (eg demographic, clinical, social) and information on exposures and potential confounders	Page 5
		(b) Indicate number of participants with missing data for each variable of interest	Page 5
		(c) Summarise follow-up time (eg, average and total amount)	Page 5
Outcome data	15*	Report numbers of outcome events or summary measures over time	Page 6-8

Main results	16	(*a*) Give unadjusted estimates and, if applicable, confounder-adjusted estimates and their precision (eg, 95% confidence interval). Make clear which confounders were adjusted for and why they were included	Page 10-11
		(*b*) Report category boundaries when continuous variables were categorized	Page 9
		(*c*) If relevant, consider translating estimates of relative risk into absolute risk for a meaningful time period	N.A.
Other analyses	17	Report other analyses done—eg analyses of subgroups and interactions, and sensitivity analyses	Page 7-8
**Discussion**			
Key results	18	Summarise key results with reference to study objectives	Page 11-13
Limitations	19	Discuss limitations of the study, taking into account sources of potential bias or imprecision. Discuss both direction and magnitude of any potential bias	Page 13-14
Interpretation	20	Give a cautious overall interpretation of results considering objectives, limitations, multiplicity of analyses, results from similar studies, and other relevant evidence	Page 11-13
Generalisability	21	Discuss the generalisability (external validity) of the study results	Page 13-14
**Other information**			
Funding	22	Give the source of funding and the role of the funders for the present study and, if applicable, for the original study on which the present article is based	Page 14

*Give information separately for exposed and unexposed groups.

Note: An Explanation and Elaboration article discusses each checklist item and gives methodological background and published examples of transparent reporting. The STROBE checklist is best used in conjunction with this article (freely available on the Web sites of PLoS Medicine at http://www.plosmedicine.org/, Annals of Internal Medicine at http://www.annals.org/, and Epidemiology at http://www.epidem.com/). Information on the STROBE Initiative is available at http://www.strobe-statement.org.

Supplementary Table S2. Baseline characteristics stratified by bio-naïve versus bio-experienced status.

**Variables**	**Bio-naïve (N=66)**	**Bio-experienced (N=29)**	**P-value**
**Age (years)**	29 (22-43)	38 (31-54)	0.08
**Sex, male**	42 (63.6)	18 (62.1)	0.88
**Disease duration (years)**	1 (0.4-2)	4 (2-7)	**0.0001**
**Age at diagnosis†**			
A1	4 (6.1)	3 (10.3)	
A2	45 (68.2)	16 (55.2)	0.45
A3	17 (25.7)	10 (34.5)	
**Location†**			
L1 ileal	8 (12.1)	3 (10.4)	
L2 colonic	15 (22.7)	9 (31.0)	0.69
L3 ileocolonic	43 (65.2)	17 (58.6)	
**Upper GI tract**	4 (6.1)	1 (3.4)	>0.99
**Behaviour †**			
B1	37 (56.1)	8 (27.6)	
B2 stricturing	24 (36.4)	18 (62.1)	**0.04**
B3 penetrating	5 (7.5)	3 (10.3)	
**Perianal disease**	26 (39.4)	11 (37.9)	>0.99
**Prior surgery**			
Perianal	14 (21.2)	5 (17.2)	0.08
Intestinal	6 (9.1)	9 (31.0)	
**Extra intestinal manifestations**	7 (10.6)	3 (10.3)	
**Previous steroid used**	2 (3.0)	6 (20.7)	
**Prior anti-TNF**			
≥ 1	NA	21 (72.4)	
≥ 2	NA	2 (6.9)	
**Prior Vedolizumab**	NA	4 (13.8)	
**Concomitant medication with UST**			
Mesalazine	37 (56.1)	14 (48.3)	
Azathioprine	1 (1.5)	0 (0)	
**Disease activity**			
HBI	8 (7-11)	8 (7-11)	0.86
CRP (mg/l)	23.2 (8.8-56.4)	10.8 (4.8-34)	0.09
SES-CD	9 (7-12)	10.5 (8-14)	0.55
**Other tests**			
WBC	7.0 (5.2-9.5)	6.5 (4.9-8.2)	0.09

Hb	11.4 (9.9-12.5)	12.1 (9.8-13.8)	0.41
PLT	325 (237-365)	256 (208-326)	0.09
Alb	37.7 (32.9-41)	37.9 (31.9-40.2)	0.49
ESR	34 (16-51)	22 (10-37)	0.07

† Phenotypes were categorized using the Montreal classification.
The values are described as number (%) or median (IQR).
Alb, Albumin; CRP, C-reactive protein; ESR, Erythrocyte Sedimentation Rate; EIM, Extra-Intestinal Manifestation; fCal, Fecal calprotectin; Hb, Haemoglobin; HBI, Harvey–Bradshaw Index; PLT, Platelets; SES-CD, Simple Endoscopic Score for Crohn's Disease; WBC, White Blood Cells, N/A, Not Applicable

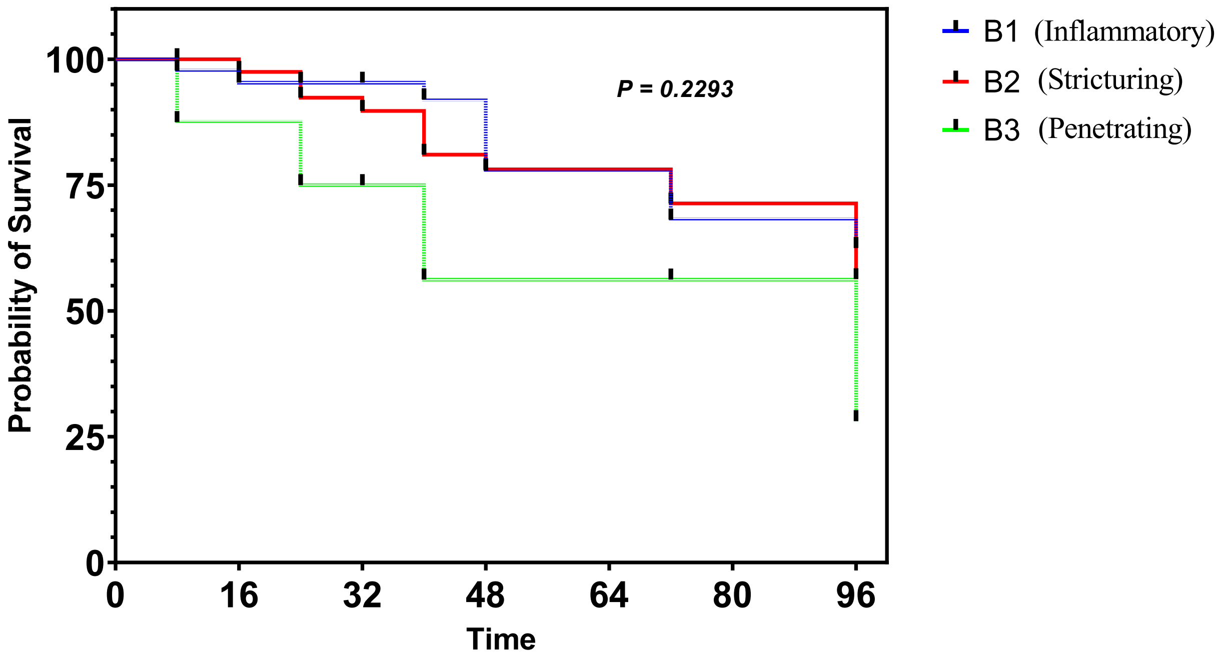

Supplementary Figure S1. Kaplan-Meier curves for treatment persistence stratified by disease behavior

Kaplan-Meier curves for treatment persistence stratified by disease behavior (B1 inflammatory, B2 stricturing, B3 penetrating) over 96 weeks showed a numerical trend toward higher persistence in B1 patients compared to B2 and B3. However, this difference was not statistically significant (*P = 0.2293*). The Log-rank test for trend also did not show a significant ordered association across the three phenotypes (*P = 0.1815*), indicating that disease behavior did not significantly influence long-term drug survival in this cohort.
